# Flavonoids in Decorticated Sorghum Grains Exert Antioxidant, Antidiabetic and Antiobesity Activities

**DOI:** 10.3390/molecules25122854

**Published:** 2020-06-20

**Authors:** Fred Kwame Ofosu, Fazle Elahi, Eric Banan-Mwine Daliri, Su-Jung Yeon, Hun Ju Ham, Joong-Hark Kim, Sang-Ik Han, Deog-Hwan Oh

**Affiliations:** 1 Department of Food Science and Biotechnology, College of Agriculture and Life Sciences, Kangwon National University, Chuncheon, Gangwon-do 24341, Korea; fkofosu17@gmail.com (F.K.O.); elahidr@gmail.com (F.E.); ericdaliri@yahoo.com (E.B.-M.D.); 2 Department of Food Science and Biotechnology, Kangwon Institute of Inclusive Technology, Kangwon National University; Chuncheon, Gangwon-do 24341, Korea; sujung0811@gmail.com; 3 Department of Biological Environment, College of Agriculture and Life Sciences, Kangwon National University, Chuncheon, Gangwon-do 24341, Korea; widehamboo@kangwon.ac.kr; 4 Department of Medical Biotechnology, College of Biomedical Sciences, Kangwon National University, Chuncheon, Gangwon-do 24341, Korea; jhkim2@erom.co.kr; 5 Department of Southern Area Crop Science, NICS Upland Crop Breeding Res. Div., 181, Hyeoksin-ro, Iseo-myeon, Wanju-Gun, Jeollabuk-do 55365, Korea; han0si@korea.kr

**Keywords:** sorghum grains, decortication, digestive enzymes inhibitors, flavonoids, advanced glycation end products, functional food

## Abstract

Eight new genotypes of brown sorghum grain were decorticated and assessed for their antioxidant, antidiabetic and antiobesity activities in vitro. The DPPH and ABTS radical scavenging assays of the soluble fractions were evaluated, followed by digestive enzymes and advanced glycation end-products (AGEs) formation inhibition assays. DSOR 33 and DSOR 11 exhibited the highest DPPH (IC_50_ = 236.0 ± 1.98 µg/mL and 292.05 ± 2.19 µg/mL, respectively) and ABTS radical scavenging activity (IC_50_ = 302.50 ± 1.84 µg/mL and 317.05 ± 1.06 µg/mL, respectively). DSOR 17, DSOR 11 and DSOR 33 showed significantly higher inhibitory activity of both α-glucosidase and α-amylase (IC_50_ = 31.86, 35.10 and 49.40 µg/mL; and 15.87, 22.79 and 37.66 µg/mL, respectively) compared to acarbose (IC_50_ = 59.34 and 27.73 µg/mL, respectively). Similarly, DSOR 33, DSOR 11 and DSOR 17 showed potent inhibition of both AGEs and lipase with IC_50_ values of 18.25, 19.03 and 38.70 µg/mL; and 5.01, 5.09 and 4.94 µg/mL, respectively, compared to aminoguanidine (52.30 µg/mL) and orlistat (5.82 µg/mL). Flavonoids were the predominant compounds identified, with flavones being the major subclass in these three extracts. Our findings suggest that decorticated sorghum grains contain substantial amounts of flavonoids and could be promising candidates for the prevention and treatment of diabetes and obesity.

## 1. Introduction

Sorghum (*Sorghum bicolor* (L.) Moench) is one of the leading cereal crops, ranked fifth after wheat, maize, rice and barley. Sorghum is cultivated globally as food for both animal and human consumption [1]. Sorghum grains are unique in that they contain diverse phytochemicals, particularly polyphenols, which are known to significantly impact human health. These phytochemicals are mostly concentrated in the bran fraction (pericarp, testa and aleurone tissues) and are reported to be beneficial in the prevention of metabolic syndromes such as type 2 diabetes, obesity, hypertension and certain cancers [2,3]. However, the processing of cereal grains generally involves the removal of the exterior layer that envelops the grain. Decortication is the partial or complete separation of the pericarp and germ from the endosperm. The coarse, unpleasant and anti-nutritional factors present in the pericarp, which affects product quality, are removed by this method. Furthermore, decortication also substantially reduces the content of bioactive compounds such as polyphenols, which are concentrated in the outer layers of the grain [4,5]. Thus, there are concerns that decorticated grains used for various foods and beverages may not have adequate amounts of phytochemicals necessary to exert biological activities that promotes human health.

Type 2 diabetes (T2D) is characterized by hyperglycemia and abnormal carbohydrate metabolism, developed due to insulin resistance and pancreatic β-cell dysfunction [6,7]. The overproduction of reactive oxygen species (ROS) has been associated with chronic exposure to high blood glucose levels. Diabetes progression is related to excessive ROS-induced oxidative stress causing damage to cells resulting in dysregulation of several gene expression leading to impaired insulin secretion and signaling [6,8,9]. The onset and development of T2D has been correlated with postprandial blood glucose levels [10]. The formation of advanced glycation end-products (AGEs) occurs as a result of excessive non-enzymatic glycation of proteins by sugars, which further complicates diabetes by contributing to the pathogenesis of cardiovascular diseases [11]. Obesity, on the other hand, is characterized by excessive adipose accumulation with impaired lipid and carbohydrate metabolism strongly associated with the pathogenesis of chronic diseases such as T2D, cardiovascular diseases and certain cancers. The prevalence of obesity and diabetes is a global health problem which has received increased attention over the past two decades [12]. α-glucosidase is the enzyme that catalyzes the key step of carbohydrate digestion and glucose release. Likewise, intestinal lipases are involved in the digestion of lipids. Therefore, the inhibition of digestive enzymes (α-glucosidase, α-amylase and pancreatic lipase) is one of the effective therapeutic strategies for controlling postprandial hyperglycemia by retarding the absorption of glucose and preventing obesity by reducing intestinal fat absorption [13]. However, due to the adverse side effects of antidiabetic and antiobesity medications, the search for natural therapeutics is gaining increasing attention. The improvement of glucose and lipid metabolism as well as the reduction of pro-inflammatory biomarkers and oxidative stress in vivo by the consumption of whole sorghum or bran have revealed their potential as good sources of novel ingredients to control and treat diabetes and obesity complications [14,15,16].

Investigations on sorghum as natural inhibitors of advanced glycation formation are paucity. In addition, the contribution of phenolic compounds from decorticated sorghum grains to health promotion and disease prevention has been an issue of major concern and demands further studies. Therefore, the aim of the present study was to screen and select the eight best decorticated new genotypes of brown sorghum grain supplied by the NICS Upland Crop Breeding Division, South Korea based on antioxidant properties, digestive enzymes (α-amylase, α-glucosidase, pancreatic lipase) and AGEs inhibition activities of their ethanol extracts in vitro. The phenolic composition of extracts was identified by UHPLC-ESI-QTOF-MS^2^.

## 2. Results and Discussion

### 2.1. Total Phenolic, Flavonoid and Condensed Tannins Contents

The eight decorticated sorghum varieties were investigated for their soluble TPC, TFC and CTC (Table 1). Soluble TPC ranged from 74.79 to 137.2 mg ferulic acid equivalent/100 g, dry weight (DW). DSOR 21 had the lowest TPC (*p* < 0.05) (74.79 ± 5.90 mg FAE/100 g). However, the TPC of DSOR 03, DSOR 08, DSOR 11, DSOR 17 and DSOR 24 did not show significant variation. Generally, whole sorghum grains are reported to contain higher phenolic content compared to decorticated ones. The TPC of decorticated sorghum grains (144 mg gallic acid equivalent/100 g) reported by Moraes et al. [17] was similar to some varieties used in this study. Our findings agree with a previous study which reported the ethanol and methanol soluble TPC of ten sorghum grain varieties to be 86–134 and 76–125 mg gallic acid equivalents/ 100 g grain, respectively [18]. Other studies have also reported a significantly higher TPC for whole sorghum compared to those found in this work [19,20,21]. Flavonoids constituted the main phenolic class in the free phenolic fraction identified in this study (Table 2, Table 3 and Table 4). TFC in decorticated sorghum grain varieties ranged from 110.3 to 126.5 mg catechin equivalent/100 g, DW. There was no significant variation (*p* < 0.05) among all decorticated varieties. However, DSOR 21 and DSOR 08 showed the lowest (110.3 ± 9.30 mg CE/100 g) and highest (126.5 ± 10.42 mg CE/100 g) TFC, respectively. Nonetheless, the TFC of decorticated sorghum grains in this study was higher than those reported by Moraes et al. [17], Dia et al. [18] and Shen et al. [20]. However, the values reported here are significantly lower than those reported by Wu et al. [21]. The variations in reported values may be due to the genotype, environment and climatic conditions as well as different extraction solvent and procedure which could have considerable impact on the phenolic amount, content and type.

The present study found the CTC to vary from 56.65 to 132.50 mg catechin equivalent/100 g, DW. The highest CTC (*p* < 0.05) was observed in DSOR 17 (132.50 CE/100 g), DSOR 01 (128.87 CE/100 g) and DSOR 21 (116.40 mg CE/100 g). There was no significant variation between DSOR 08 (81.48 CE/100 g), DSOR 11 (92.95 CE/100 g) and DSOR 24 (98.56 CE/100 g). DSOR 03 and DSOR 33 showed the lowest CTC of 67.49 and 56.65 mg CE/100 g, respectively. The CTC of decorticated sorghum flour reported by Moraes et al. [17] was comparatively lower (54.00 mg CE/100 g) than those found in this study, although two varieties were in agreement [22]. In contrast with those reported by other studies, the values reported herein are significantly lower [19,20]. Tannin free sorghum was reported to have tannin content ranging from 50–380 mg/100 g grain [23]. Thus, the findings in this work suggest this sorghum flour as tannin free.

### 2.2. Antioxidant Properties

**Figure 1:**
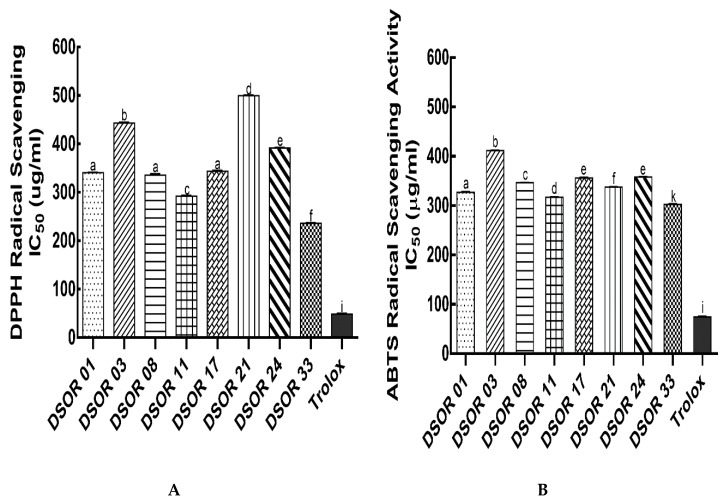
Antioxidant activities of soluble extracts of decorticated sorghum varieties. (**A**) 2,2′-diphenyl-1-picrylhydrazyl (DPPH) radical scavenging activity IC_50_ values; (**B**) 2,2′-azino-bis (3-ethylbenzothiazoline-6-sulfonic acid) diammonium salt (ABTS) radical scavenging activity IC_50_ values. Different lowercase letters denote significant difference (*p* < 0.05).

The antioxidant property of sorghum grains has been attributed to their phenolic compounds [14,15,16]. DPPH and ABTS radical scavenging assays were utilized to assess the antioxidant capacity of the different decorticated sorghum grain extracts (Figure 1A,B). The DPPH radical scavenging activity was highest in DSOR 33 (IC_50_ value = 236.0 ± 1.98 µg/mL), followed by DSOR 11 (IC_50_ value = 292.05 ± 2.19 µg/mL). However, no significant difference (*p* < 0.05) was observed among DSOR 01, DSOR 08 and DSOR 17 (IC_50_ values = 340.70 ± 1.13, 335.75 ± 3.18 and 343.85 ± 1.77 µg/mL, respectively). DSOR 24, DSOR 03 and DSOR 21 showed the lowest DPPH radical scavenging activity with IC_50_ values of 391.80 ± 1.27, 443.3 ± 2.12 and 499.75 ± 1.49 µg/mL, respectively, compared to standard antioxidant compound, Trolox (48.7 ± 2.19 µg/mL). Likewise, DSOR 33 and DSOR 11 exhibited the highest ABTS radical scavenging activity with IC_50_ values of 302.50 ± 1.84 and 317.05 ± 1.06 µg/mL, respectively. The soluble extracts of DSOR 01, DSOR 21, DSOR 08, DSOR 17, DSOR 24 and DSOR 03 had a significantly lower ABTS radical scavenging activity with IC_50_ values of 327.15 ± 1.77, 337.65 ± 1.06, 346.85 ± 0.35, 356.10 ± 1.56, 358.35 ± 1.07 and 411.65 ± 1.20 µg/mL, respectively, compared to Trolox (74.60 ± 1.45 µg/mL). This followed a similar pattern as observed with the DPPH assay. It should be noted that, although several studies report that decortication decreases the antioxidant activity (expressed as mg or µM Trolox equivalents g^−1^ or mg ascorbic acid equivalents g^−1^) in sorghum grains, no literature showing activity expressed as IC_50_ value was found [19,20]. Hence, it was hard to compare with such results. Notwithstanding, these results show high antioxidant activity by some decorticated sorghum grain varieties and their potential to be developed as functional ingredients.

### 2.3. Digestive Enzymes and Glycation Formation Inhibition

**Figure 2:**
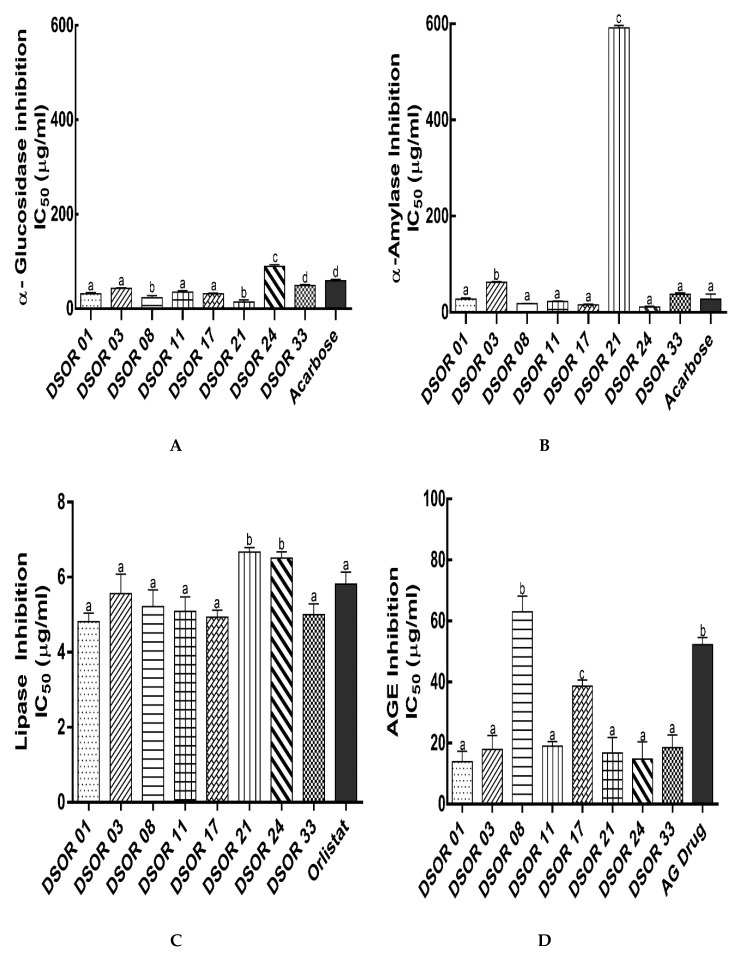
Digestive enzymes and advanced glycation end-products inhibitory activities from ethanol extracts of decorticated sorghum varieties. (**A**) α-glucosidase inhibitory activity IC_50_ values; (**B**) α-amylase inhibitory activity IC_50_ values; (**C**) lipase inhibitory activity IC_50_ values and (**D**) AGEs inhibitory activity IC_50_ values. AG, aminoguanidine. Different lowercase letters denote significant difference (*p* < 0.05).

Whole sorghum flour or bran consumption has been reported to improve glucose and lipid metabolism by inhibiting fat accumulation, pro-inflammatory biomarkers and increased the antioxidant status in animal and some clinical human studies [14,15,16,24]. One of the primary preventive strategies in the management of T2D and obesity is the retardation of carbohydrate and fat digestion and absorption to reduce postprandial hyperglycemia and lipid accumulation. Thus, the inhibition of digestive enzymes (α-amylase, α-glucosidase and pancreatic lipase) and AGE formation by decorticated sorghum grain extracts was evaluated (Figure 2A–D).

**Figure 3:**
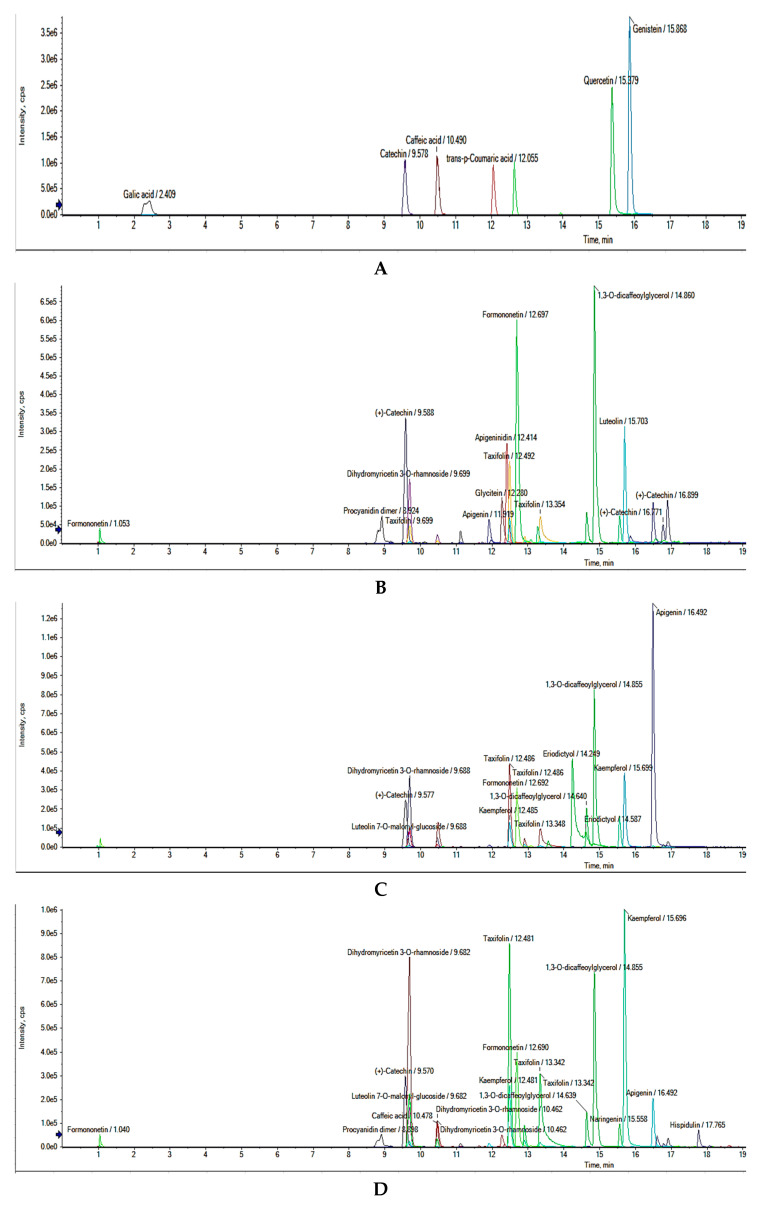
Decorticated sorghum grain phenolic compounds chromatographs. (**A**) Authentic standard compounds; (**B**) DSOR 11; (**C**) DSOR 17 and (**D**) DSOR 33. Detection was achieved at 280 nm.

DSOR 21 and DSOR 08 ethanol extracts showed the highest inhibition of α-glucosidase with IC_50_ values of 14.04 ± 3.60 and 23.31 ± 4.56 µg/mL, followed by DSOR 17, DSOR 01, DSOR 11 and DSOR 03 with IC_50_ values of 31.86 ± 1.37, 31.96 ± 2.17, 35.10 ± 3.10 and 43.12 ± 1.38 µg/mL, respectively, which were significantly effective with respect to the standard drug, acarbose IC_50_ value (59.34 ± 3.07 µg/mL). Moreover, DSOR 33 had a strong α-glucosidase inhibition with IC_50_ value of 49.40 ± 1.88 µg/mL significantly potent as acarbose (59.34 ± 3.07 µg/mL) (Figure 2A). DSOR 24 soluble extracts exhibited the lowest α-glucosidase inhibition with IC_50_ value of 89.51 ± 3.61 µg/mL among all tested decorticated sorghum grains. Six sorghum ethanolic extracts were found to inhibit α-glucosidase with IC_50_ values ranging from 1.1–102.7 µg/mL. Four of these extracts showed significantly higher inhibition (1.1–1.4 µg/mL) compared to those reported in this work. This effect was attributed to phenolic compounds, although authors did not identify and characterize them [25]. Lower inhibitory activities in the present study could be attributed to the decortication. There was no significant variation in terms of α-amylase inhibition exhibited by DSOR 24, DSOR 17, DSOR 08, DSOR 11, DSOR 01, DSOR 33 and acarbose with IC_50_ values of 11.21 ± 1.58, 15.87 ± 1.14, 18.12 ± 0.83, 22.79 ± 1.05, 27.64 ± 2.41, 37.66 ± 2.92 and 27.73 ± 7.34 µg/mL, respectively. However, DSOR 03 and DSOR 21 showed the lowest inhibition of α-amylase with IC_50_ values of 62.88 ± 1.51 and 591.96 ± 4.87 µg/mL, respectively (Figure 3B). Some of the results in this study corroborate recent studies which reported strong α-glucosidase and α-amylase inhibitory activities (IC_50_ values) of 10.78 ± 0.63 and 16.93 ± 1.08 µg/mL, respectively, from red sorghum methanol extracts [26]. As mentioned earlier, the genotype, environmental conditions, extraction solvent and process may affect the type, content of phenolic compounds and hence their inhibitory activities.

Flavonoids have shown inhibitory properties against digestive enzymes. Flavonoids constituted the majority of phenolic compounds identified in soluble extracts (Table 2, Table 3 and Table 4). Flavones, the predominant flavonoid in DSOR 17 and DSOR 33 have C2=C3 double bond and may account for its bioactive potency against T2D related enzymes (Table 3 and Table 4). Our results corroborate the findings from Lim et al. [27] which delineated the significance of the hydroxyl group at C3 of the C-ring and double bond between C2 and C3 on the C-ring of flavonoids in α-glucosidase and α-amylase inhibition, respectively. Emerging evidence suggests that low polyphenol grains with specific bioactive monomeric flavone composition may have major impact on disease prevention than high polyphenol grains. However, synergistic effects with other flavonoids could enhance inhibitory activities. The mechanism by which phenolic compounds inhibit digestive enzymes are attributed with their binding to amino acid residues at enzyme active sites via hydrogen bonding, thereby inhibiting the catalytic reaction of enzymes [28,29,30]. Results from this work indicate that decorticated sorghum grains contain substantial flavonoids which could retard carbohydrate hydrolyzing enzymes and thus help ameliorate diabetes and its associated diseases.

The formation of advanced glycation end-products (AGEs) are linked to the onset and progression of diabetes. Polyphenols have been shown to exert anti-glycation effects (Yilmaz et al., 2018; Yeh et al., 2017; Crascì et al., 2018). In comparison with anti-glycation drug, aminoguanidine (IC_50_ = 52.30 ± 2.31 µg/mL), the soluble extracts of DSOR 01, DSOR 03, DSOR 11, DSOR 21, DSOR 24 and DSOR 33 exhibited the strongest inhibition of AGEs formation with IC_50_ values of 13.93 ± 3.47, 17.97 ± 4.56, 19.03 ± 1.49, 16.79 ± 5.09, 14.77 ± 5.69 and 18.52 ± 4.19 µg/mL, respectively. DSOR 17 showed moderately strong AGEs inhibition (IC_50_ = 38.70 ± 2.20 µg/mL). Although, DSOR 08 extracts showed significantly lower inhibition of AGEs formation with IC_50_ value of 63.00 ± 5.25 µg/mL, its potency was significantly comparable to the drug, aminoguanidine (IC_50_ = 52.30 ± 2.31 µg/mL) (Figure 2D). Farrar et al. [31] demonstrated that ethanolic extracts of certain sorghum bran varieties which contained the anthocyanins, luteolinidin and apigeninidin significantly inhibited protein glycation. These brans also showed high phenolic content and antioxidant activity compared to other cereal brans (oat, rice and wheat). The phenolic content and antioxidant activity of polyphenols have shown strong correlation with protein glycation inhibition [32]. Our findings agree with previous studies which demonstrated significant protein glycation inhibition by flavonoids.

Flavonoids have been reported to exert antiobesity actions both in vitro and in vivo by suppressing triglyceride accumulation, reducing adipocytes viability and proliferation, stimulating lipolysis, β-oxidation of fatty acids, and reducing inflammation [33]. In the present work, we assessed pancreatic lipase inhibition by soluble extracts of decorticated sorghum grains to explore their efficacy in preventing obesity by reducing intestinal fat absorption. Six out of the eight (DSOR 01, DSOR 17, DSOR 33, DSOR 11, DSOR 08 and DSOR 03) decorticated sorghum grain extracts showed potent lipase inhibition with IC_50_ values of 4.82 ± 0.23, 4.94 ± 0.17, 5.01 ± 0.28, 5.09 ± 0.39, 5.22 ± 0.45, and 5.56 ± 0.52 µg/mL, respectively, comparable to the drug Orlistat (5.82 ± 0.32 µg/mL). DSOR 21 and DSOR 24 exhibited a slightly lower lipase inhibition with IC_50_ values of 6.70 ± 0.12 and 6.51 ± 0.17 µg/mL, respectively, compared to Orlistat and other tested decorticated sorghum grain extracts (Figure 2C). Evidence from several in vitro, in vivo and some human studies have revealed that flavonoids enhance ROS scavenging activities, interaction and inhibition of carbohydrate and fat catabolism enzymes, thus decreasing fat accumulation and, therefore, preventing obesity [34,35,36].

### 2.4. Correlation Analysis of Total Phenolics, Antioxidant Capacity and Digestive Enzymes Activities

Besides their antioxidant properties, polyphenols could interact and inhibit enzyme activities. The correlations between TPC, DPPH and ABTS radical scavenging activity, α-amylase, α-glucosidase and lipase activity are shown in supplementary Table S1. TPC showed strong negative correlation with DPPH radical scavenging (*r* = −0.77; *p* = 0.02) and α-amylase (*r* = −0.65; *p* = 0.08) activity. Similarly, good negative correlation was observed between TPC and ABTS (*r* = −0.47; *p* = 0.24) radical scavenging and lipase (r = −0.53; *p* = 0.18) activity. However, TPC exhibited a linear correlation with α-glucosidase activity (r = 0.33; *p* = 0.42). Similar findings were reported by Shen et al. [20], who found a strong correlation between total phenolics and antioxidant activities. DPPH radical scavenging activity demonstrated good linear correlations of 0.63, 0.69 and 0.76 with ABTS radical scavenging, α-amylase and lipase activity, respectively. This corroborates studies which reported significant negative correlation between DPPH radical scavenging activity and TPC, and linear relationship with α-amylase [37]. However, no significant correlation was observed for α-glucosidase and AGE. α-amylase and α-glucosidase activity exhibited linear correlation of 0.66 and 0.28, respectively with lipase activity. The type and content of individual phenolic compounds have been found to play significant roles in antioxidant and enzyme inhibitory activities [20,37]. Thus, correlation analysis of ethanol extracts of decorticated sorghum grains shows the potential role of polyphenols in scavenging free radicals, resulting in enzyme inhibition.

### 2.5. Untargeted UHPLC-Q-TOF-MS^2^ Phenolic Compounds Identification

As phytotherapeutics, phenolic compounds are known for their health-promoting properties and prevention of chronic diseases. Untargeted phenolic profiling of selected decorticated sorghum soluble extracts (DSOR 11, DSOR 17 and DSOR 33) based on their potent antioxidant, antidiabetic and antiobesity properties (α-amylase, α-glucosidase, lipase, AGEs inhibition) were positively or tentatively identified using UHPLC-Q-TOF-MS^2^. UHPLC phenolic compounds profile in decorticated sorghum extracts are shown in Figure 3. Mass spectra information of all identified phenolic compounds are provided as supplementary materials. The mass spectra data are summarized in Table 2, Table 3 and Table 4. Phenolic compounds were identified and characterized by comparing retention time (RT) with available authentic standards and confirmed by UHPLC-Q-TOF-MS^2^. Compounds were tentatively identified with a comprehensive online polyphenol database, Phenol Explorer [38], and comparison with other mass spectra literature evidence [20,39,40]. Table 2, Table 3 and Table 4 show a total of twelve (12), thirteen (13) and eighteen (18) phenolic compounds positively and tentatively identified in soluble extracts of DSOR 11, DSOR 17 and DSOR 33, respectively. Flavonoids were the predominant phenolic compounds identified in these extracts, with majority existing as aglycones and only two as glycosides in DSOR 17 and DSOR 33 (dihydromyricetin 3-*O*-rhamnoside and luteolin 7-*O*-malonyl-glucoside). Dihydromyricetin 3-*O*-rhamnoside was the only glycoside present in DSOR 11.

Isoflavones (glycitein, formononetin), flavanols (procyanidin dimer, catechin), flavones (apigenin, luteolin) constituted the majority of flavonoids in DSOR 11; however, other sub-classes were present, including 3-deoxyanthocyanidins (apigeninidin), dihydroflavonols (taxifolin, dihydromyricetin 3-*O*-rhamnoside) and flavanone (naringenin). 1,3-*O*-dicaffeoylglycerol, a derivative of caffeic acid, was the only phenolic hydroxycinnamic acid present. The predominant flavonoid sub-class in DSOR 17 and DSOR 33 was flavones (apigenin, luteolin, hispidulin, luteolin 7-*O*-malonyl-glucoside). These flavones were mostly aglycones or *O*-glycosides, whereas C-glycosides have been reported in other cereal grains. This is in agreement with available literature [41,42]. Our finding corroborates previous studies, which reported apigenin and luteolin as the main flavones in some sorghum genotypes [41,43]. Another study also identified these flavones in certain genotypes of sorghum [43]. Other flavonoid sub-classes present in DSOR 17 included isoflavone (formononetin), dihydroflavonols (taxifolin, dihydromyricetin 3-*O*-rhamnoside), flavanones (naringenin, eriodictyol), flavanols (catechin) and flavonol (kaempferol). Similarly, in addition to the predominant flavones in DSOR 33, different flavonoid groups present included isoflavones (formononetin, glycitein), flavanonol (taxifolin), flavanols (procyanidin dimer, catechin), dihydroflavonols (dihydromyricetin 3-*O*-rhamnoside), flavonol (kaempferol) and flavanone (naringenin). This finding is consistent with an earlier report which indicates flavones as the dominant monomeric flavonoid in sorghum and other cereal grains [42]. Other studies have also already reported the flavonoids naringenin, eriodictyol, catechin, and taxifolin in sorghum methanolic extracts [19,20,40,44]. Although, 1,3-*O*-dicaffeoylglycerol was present in all three extracts, caffeic acid was only identified in DSOR 17 and DSOR 33, but not in DSOR11. Furthermore, glycitein and procyanidin dimer were present in DSOR 11 and DSOR 33, but not in DSOR 17. Additionally, apigeninidin, a 3-deoxyanthocyanidin which is specific to sorghum grains, was only found in DSOR 11.

Available standards used for positive identification included gallic acid, catechin, caffeic acid, *p*-coumaric acid, ferulic acid and quercetin. Compound 2 ([M−H]^−^ *m/z* = 289.0721) was positively identified as catechin when compared with catechin standard as shown in Table 2. All other phenolics were tentatively identified by comparing with mass spectra literature data and an online polyphenol database. Furthermore, compounds 4 and 12 showed the same [M−H]^−^ at *m/z* 269.0459, suggesting the possibility of being an isomeric pair. Therefore, compounds 4 and 12 were tentatively identified as apigenin. Likewise, in Table 3, compound 2 ([M−H]^−^ *m/z* = 289.0721) was positively identified as catechin by comparing with the catechin standard. Compounds 4 and 6 showed the same [M−H]^−^ at *m/z* 303.0513, suggesting the possibility of being an isomeric pair. Thus, compounds 4 and 6 were tentatively identified as taxifolin. Consequently, compounds 7 and 12 were tentatively identified as kaempferol and luteolin, respectively, as they showed the same [M−H]^−^ at *m/z* 285.0406, indicating the possibility of being an isomeric pair. From Table 4, compound 2 ([M−H]^-^ *m/z* = 289.0721) was positively identified as catechin by comparing with the catechin standard. Compounds 3 and 7 showed the same [M−H]^−^ at *m/z* 465.1044, suggesting the possibility of being an isomeric pair. Thus, compounds 3 and 7 were tentatively identified as dihydromyricetin 3-*O*-rhamnoside. Furthermore, compounds 5, 9 and 12 were tentatively identified as taxifolin with the same [M−H]^−^ at *m/z* 303.0513, suggesting the possibility of being an isomeric pair. Furthermore, compounds 10 and 15 showed the same [M−H]^−^ at *m/z* 285.0406, indicating that they could possibly be an isomeric pair. Consequently, compounds 10 and 15 were tentatively identified as kaempferol and luteolin, respectively. Compounds 17 and 18 had the same [M−H]^−^ at *m/z* 299.0563, suggesting their possibility of being an isomeric pair. Consequently, compounds 17 and 18 were tentatively identified as hispidulin. To the best of our knowledge, we report for the first time the presence of glycitein, formononetin and hispidulin in decorticated sorghum grains, although these flavonoids have only been reported in fruits, vegetables and legumes.

Table 5 summarizes the quantitative analysis using available phenolic standards by HPLC-PDA. Phenolic compounds were quantified by comparing with the calibration curves of phenolic standards (gallic acid, caffeic acid, ferulic acid, *p*-coumaric acid, catechin, quercetin and genistein). The catechin content of DSOR 11, DSOR 17 and DSOR 33 were found to be 103.03 µg/100 g, 83.55 µg/100 g and 140.99 µg/100 g, respectively. This may account for the higher inhibition activities of T2D related digestive enzymes as catechins have been reported for their ameliorating effects in diabetic animals [43,45]. However, the diversity of flavonoids present may also synergistically contribute to enhance the antidiabetic and antiobesity effect. Moreover, caffeic acid was absent in DSOR 11, but present in DSOR 17 (44.93 µg/100 g) and DSOR 33 (49.82 µg/100 g). Previous studies also reportedly identified caffeic acid in the free form of sorghum grain extracts [19,20,40,46]. The content of caffeic acid (1.14–3.81 mg/100 g) in free extracts of eight whole sorghum grain varieties was higher than reported in this study [20]. The catechin content were almost twofold and threefold higher than caffeic acid content in DSOR 17 and DSOR 33, respectively. The remaining four phenolic standards were not present in the soluble fractions of DSOR 11, DSOR 17 and DSOR 33. Our findings are consistent with previous reports that phenolic acids in cereals are mostly present in bound form than in free fractions [47].

## 3. Materials and Methods

### 3.1. Chemicals

The phenolic standards—caffeic acid, gallic acid, ferulic acid, *p*-coumaric acid, catechin, quercetin and genistein were purchased from Sigma Aldrich (Seoul, Korea). The enzymes α-glucosidase from *Saccharomyces cerevisiae,* α-amylase from *Bacillus licheniformis* (Chicago, IL, USA), pancreatic lipase, acarbose, Orlistat, aminoguanidine (AG), 4-methylumbelliferyl oleate (4-MU) and 2-methoxyethanol were all purchased from Sigma Aldrich (Seoul, Korea). Analytical grade reagents including potassium phosphate monobasic, potassium phosphate dibasic, sodium dihydrogen phosphate, sodium phosphate dibasic, sodium chloride, sodium carbonate, soluble starch, sodium hydroxide, 3,5-dinitrosalicyclic acid, sodium citrate, 4-nitrophenyl α-d-glucopyranoside, 2,2′-diphenyl-1-picrylhydrazyl (DPPH), Folin–Ciocalteu reagent, 2,2′-azino-bis (3-ethylbenzothiazoline-6-sulfonic acid) diammonium salt (ABTS), 6-hydroxy-2,5,7,8-tetramethylchromane-2-carboxylic acid (Trolox), ethanol, hexane, methanol, hydrochloric acid, potassium persulfate and sodium carbonate were purchased.

### 3.2. Sample Preparation

**Figure 4:**
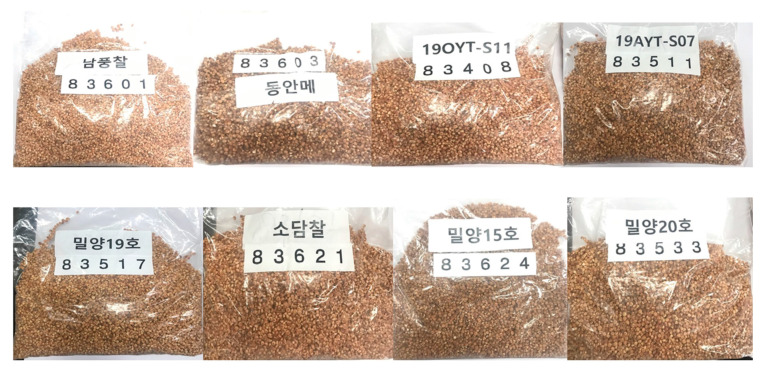
Pictures of decorticated sorghum grains.

Decorticated sorghum grain varieties used in this work were provided by Korean Rural Development Administration (KRDA), Jeonju, South Korea. Decorticated grains were labeled DSOR 01, DSOR 03, DSOR 08, DSOR 11, DSOR 17, DSOR 21, DSOR 24 and DSOR 33 (Figure 4). The grains were cleaned and ground into fine powder using an electric mill and sieved through mesh 40. Samples were kept at −20 °C until further analysis.

#### 3.2.1. Extraction of Polyphenols

The procedure by Pradeep and Sreerama [48] was used for soluble or free phenolic compounds extraction with some modification. Samples were first defatted by hexane using Soxhlet apparatus. Defatted samples (5 g) were extracted with 70% ethanol (1:20 *w*/*v*) in an orbital shaker for 1 h at 50 °C. The supernatant was collected and the residue was re-extracted twice under the same conditions after centrifuging at 4000× *g* for 10 min. Supernatants were combined and concentrated under vacuum at 40 °C and then freeze-dried. Lyophilized extracts were stored at −20 °C and later reconstituted in ethanol for further analysis.

#### 3.2.2. Total Phenolic Content (TPC)

The method described by Ainsworth and Gillespie [49] was used for total phenolic content (TPC) measurement with slight modification using a 24-well microplate. Ferulic acid was used as the standard. Briefly, one of the 100 μL sample extracts, the standard or the 95% (*v*/*v*) methanol blank was added to 200 μL Folin–Ciocalteu reagent and vortex thoroughly. After adding 800 μL of 700 mM sodium carbonate, the mixture was incubated at room temperature for 2 h. The absorbance was read at 765 nm using the SpectraMax i3 plate reader (Molecular Devices Korea, LLC, Seoul, Korea. Using the ferulic acid standard curve, the total phenolic content was calculated and expressed as milligrams of ferulic acid equivalents per 100 g of sample (mg FAE/100 g).

#### 3.2.3. Total Flavonoid Content (TFC)

Using the AlCl_3_ method described by Apea-Bah et al. [50], the total flavonoid content (TFC) of ethanol extracts was determined with some modifications. Briefly, one of the 250 μL sample extracts or the standard was added to 75 μL NaNO_2_ (50 g L^−1^) and 1 mL distilled water. After 5 min, 75 μL AlCl_3_ (100 g L^−1^) was added to the reaction mixture. Then, 500 μL of 1 M NaOH and 600 μL distilled water was added after 6 min. The absorbance was read at 510 nm after shaken for 30 s in SpectraMax i3 plate reader (Molecular Devices Korea, LLC, Seoul, Korea). Catechin was used as the standard and results were expressed as milligram catechin equivalents per 100 g of sample (mg CE/100 g).

#### 3.2.4. Total Condensed Tannin Content (CTC)

The vanillin assay method with modification was used for total condensed tannin content (CTC) measurement [51]. Briefly, sample solution (40 μL) was mixed with 4% vanillin reagent (200 μL) in a 96-well microplate. The absorbance at 500 nm was read after the mixture was maintained at 30 °C for 20 min against a blank solution (4% vanillin reagent substituted with 4% hydrochloric acid). Catechin was used as the standard and results were expressed as milligrams of catechin equivalents per gram of sample (mg CE/100 g) on dry weight basis.

#### 3.2.5. DPPH Radical Scavenging Activity

The method previously described by Ofosu et al. [52] was used to measure DPPH radical scavenging by ethanol extracts using a 24-well microplate reader. Briefly, 200 μL sample extracts of various concentrations were added to 2 mL freshly made DPPH radical solution (100 μM). After 30 min incubation at room temperature, absorbance was read at 517 nm. The results were expressed in terms of IC_50_ representing the concentration of test extracts required to scavenge DPPH free radical by 50%.

#### 3.2.6. ABTS Radical Scavenging Activity

The ABTS radical scavenging assay described by Ofosu et al. [52] was used. ABTS^+^ stock solution was prepared by reacting equal volumes of 7 mM ABTS solution with 2.45 mM potassium persulfate solution in the dark at room temperature for 16 h. Using ethanol, the ABTS+ stock solution was diluted to obtain an absorbance of approximately 0.70 at 734 nm. Appropriately, 80 μL sample extracts of various concentrations were added to 1 mL freshly prepared ABTS+ radical solution, and the absorbance was read at 734 nm. The results were expressed in terms of IC_50_ representing the concentration of test extracts required to scavenge ABTS radical by 50%.

#### 3.2.7. α-Amylase Inhibitory Assay

The α-amylase inhibitory assay described by Sekhon-Loodu and Rupasinghe [37] was used with some modification. The test extracts, enzyme and soluble starch were dissolved in 20 mM sodium phosphate buffer containing 6 mM NaCl (pH 6.9). Pancreatic porcine α-amylase (250 μL) (1 U/mL, dissolved in the buffer at pH 6.9) was added to 100 μL of test extract at different concentrations. The mixture was pre-incubated at 37 °C for 15 min, before the addition of 250 μL of 0.5% starch. The reaction mixture was terminated using 1 mL of dinitrosalicylic acid color reagent after vortexing and incubating again at 37 °C for 20 min. The tubes were placed in a heating block for 5 min, cooled to room temperature and diluted with ultrapure water. Two hundred microliters of the reaction mixture were placed into a 96-well clear plate, and the absorbance was read at 540 nm using the SpectraMax i3 plate reader (Molecular Devices Korea, LLC, Seoul, Korea). The control (α-amylase without any inhibitor) represented 100% enzyme activity. Appropriate test extract blanks containing the reaction mixture except the enzyme were used to correct for the color interference. Acarbose, a known antidiabetic drug was used as a positive control. The percentage inhibition of the test sample on α-amylase was calculated as:

Inhibition (%) = 100 × (A_C_ − A_S_)/A_C_ (1)

where A_C_, A_S_ is the absorbance of control and test sample, respectively. The results were expressed in terms of IC_50_ representing the concentration of test extracts required to cause the enzyme inhibition by 50%.

#### 3.2.8. α-Glucosidase Inhibitory Assay

The α-glucosidase inhibitory assay was conducted as described by Sekhon-Loodu and Rupasinghe [37] with some modification. Various concentrations of freeze-dried extracts were dissolved in 10 mM potassium phosphate buffer (pH 6.8). 100 μL of extract at different concentrations, 100 μL a-glucosidase (0.5 U/mL) and 300 μL of 10 mM potassium phosphate buffer (pH 6.8) were added to a 24-well microplate and then pre-incubated at 37 °C for 15 min after which 100 μL of 5 mM *p*-nitrophenol-α-d-glucopyranoside substrate was added. The reaction mixture was then incubated at 37 °C for 15 min after which 400 μL of 200 mM sodium carbonate was added. The absorbance at 405 nm was measured using the SpectraMax i3 plate reader (Molecular Devices Korea, LLC, Seoul, Korea). The control was the mixture of the enzyme and substrate without inhibitors; rather, a buffer was used. The sample blanks were the mixtures of test sample, substrate and buffer except α-glucosidase. Acarbose was used as positive control. The inhibition (%) of α-glucosidase was calculated same way as α-amylase assay described earlier.

#### 3.2.9. Pancreatic Lipase Inhibition Assay

The assay used was modified according to the method described by Li et al. [53]. 4-MU oleate was used as substrate for pancreatic lipase activity. Briefly, 50 μL of lipase (50 U/mL) dissolved in phosphate buffer (200 mM, pH 7.4) were added to 50 μL of different concentrations of extracts and standard (Orlistat) in a 24-well microtiter plate and allowed to react for 10 min. After that, 100 μL of 1 mM 4-MU solution (dissolved in methyl cellosolve) was added and incubated at 25 °C for 30 min. The reaction was stopped by adding 100 μL sodium citrate solution (0.1 M, pH 4.2). The amount of 4-methylumbelliferone released was measured using a fluorescence reader at an excitation wavelength of 355 nm and an emission wavelength of 460 nm. Experiments were done in triplicates. Orlistat was used as a positive control. Percentage lipase inhibition was calculated as shown below:

(2) $$\mathrm{Lipase}\;\mathrm{Inhibition}\;\mathrm{Rate}\;(\%)=1-\left(\frac{F_{\mathrm{test}}-F_{\mathrm{test}\;\mathrm{blank}}}{F_{\mathrm{control}}-F_{\mathrm{control}\;\mathrm{blank}}}\right)\times 100$$

where F_test_ and F_test blank_ are the fluorescent values of test samples with and without the substrate 4-MU oleate, respectively. F_control_ and F_control blank_ are the fluorescent values of control with and without the substrate 4-MU oleate, respectively. The results were expressed in terms of IC_50_ representing the concentration of test extracts required to cause the enzyme inhibition by 50%.

#### 3.2.10. Inhibition of Protein Glycation

The procedure by Sekhon-Loodu and Rupasinghe [37] was adapted with slight modification. Bovine serum albumin (BSA), d-glucose and aminoguanidine were dissolved in 0.2 M phosphate buffer saline (pH 7.4) containing sodium azide (0.02% *w*/*v*). Equal volumes containing BSA (5.0 mg/mL) and d-glucose (36 mg/mL) and the negative control or the test samples or aminoguanidine at different concentrations (0.01, 0.02, 0.04, 0.05, 0.1, 0.2, 0.5 and 1.0 mg/mL) were added to reach a final volume of 1.0 mL. Aminoguanidine, a known AGE formation inhibitor, was used as the positive control. The mixtures in Eppendorf tubes were incubated in triplicate at 37 °C for a week. Fluorescent AGEs were measured using a microplate reader at 340 nm excitation and 420 nm emission wavelengths. Experiments were conducted in triplicates. The percentage of the AGE inhibition was calculated as shown below:

(3) $$\mathrm{Inhibition}\;(\%)=\left[1-\left(\frac{\mathrm{Fluorescent}\;\mathrm{of}\;\mathrm{the}\;\mathrm{test}}{\mathrm{Fluorescent}\;\mathrm{of}\;\mathrm{control}}\right)\right]\times 100$$

The results were expressed in terms of IC_50_ representing the concentration of test extracts required to cause the inhibition of AGE formation by 50%.

#### 3.2.11. UHPLC-Q-TOF-MS/MS Phenolic Compounds Characterization

An UHPLC (SCIEX ExionLC AD system, Framingham, MA, USA) equipped with a controller, AD pump, degasser, AD autosampler, AD column oven and photodiode array (PDA) detector (ExionLC) coupled to a quadrupole time-of-flight mass spectrometer (Q-TOF-MS) (X500_R_ QTOF) was used for UHPLC and mass spectrometry analyses (LC–MS^2^). The protocol described by Xiang et al. [54] was used with some modification. The analytical column was a 100 mm × 3 mm Accucore C18 column (Thermo Fisher Scientific, Waltham, MA, USA). The sample (10 μL) was injected by an autosampler and eluted through the column with a binary mobile phase consisting of A (water containing 0.1% formic acid) and B (methanol), and the flow rate of 0.4 mL/min was used. A 25 min linear gradient was programmed as follows: 0–3.81 min, 9–14% B; 3.81–4.85 min, 14–15% B; 4.85–5.89 min, 15% B; 5.89–8.32 min, 15–17% B; 8.32–9.71 min, 17–19% B; 9.71–10.40 min, 19% B; 10.40–12.48 min, 19–26% B; 12.48–13.17 min, 26–28% B; 13.17–14.21 min, 28–35% B; 14.21–15.95 min, 35–40% B; 15.95–16.64 min, 40–48% B; 16.64–18.37 min, 48–53% B; 18.37–22.53 min, 53–70% B; 22.53–22.88 min, 70–9% B; and 22.88–25.00 min, 9% B. Phenolic compounds were identified by comparing retention time (RT) and confirmed by UHPLC-Q-TOF-MS^2^. The Q-TOF-MS was calibrated for the negative mode through the mass range of 100–1000 with the resolution of 5000. Full mass spectra were recorded using a capillary voltage of 1.45 kV and a cone voltage of 30 V. The flow rates of cone gas (He) and desolvation gas (N_2_) were 45 and 900 L/h, respectively. The desolvation gas temperature was set at 250 °C, the ion source temperature was 120 °C, and the collision energies of 15, 20 and 30 V were set to acquire the MS^2^ spectra. Using the peak area at wavelengths of 280 nm for catechin and quercetin, and 320 nm for caffeic acid, ferulic acid, gallic acid and *p*-coumaric acid, individual phenolic compounds were quantified and expressed as µg/100 g.

#### 3.2.12. Statistical Analysis

GraphPad Prism (8.0, Graphpad Software, San Diego, USA) was used for data analysis. The mean differences of triplicate analysis were determined using one-way analysis of variance (ANOVA) followed by Tukey’s test at *p* < 0.05 significance level. The results were presented as mean ± standard deviation (SD).

## 4. Conclusions

Sorghum grains are rich sources of phytonutrients with promising health-promoting benefits for the prevention of metabolic syndromes. However, there are concerns that the decortication process may significantly reduce and affect the content and bioactivity of phytochemicals, which are largely present in the outer layers. This study evaluated the antioxidant, antidiabetic, antiobesity and antiglycation potential of eight decorticated sorghum grains. Decorticated sorghum grain ethanol extracts showed strong antioxidant activity. Decorticated sorghum grain phenolics demonstrated strong inhibition of α-glucosidase, α-amylase and pancreatic lipase enzymes comparable to commonly used drugs, suggesting their possibility to decrease postprandial hyperglycemia and fat absorption by retarding carbohydrate and fat digestion. Moreover, soluble extracts exhibited potent glycation inhibition demonstrating their potential use as antiglycation agents. Untargeted UHPLC-Q-TOF-MS/MS analysis revealed flavonoids as the predominant polyphenols. These results suggest that decorticated sorghum grains contain substantial amounts of flavonoids necessary to prevent disease and promote health. Additional studies are required to substantiate the antidiabetic and antiobesity benefits of these flavonoid-rich decorticated sorghum grains in animal models for their subsequent development as functional ingredients and foods.

## Figures and Tables

**Table 1:** Total phenolic content (TPC), total flavonoid content (TFC) and total condensed tannins content (CTC) of decorticated sorghum varieties.

Decorticated Varieties	TPC (mg Ferulic Acid Equivalent/100 g, DW)	TFC (mg Catechin Equivalent/100 g, DW)	CTC (mg Catechin Equivalent/100 g, DW)
DSOR 01	129.7 ± 17.06 ^a^	113.5 ± 9.10 ^a^	128.87 ± 4.27 ^a^
DSOR 03	101.2 ± 16.49 ^b^	111.8 ± 8.51 ^a^	67.49 ± 3.70 ^b^
DSOR 08	118.2 ± 5.05 ^b^	126.5 ± 10.42 ^a^	81.48 ± 4.02 ^c^
DSOR 11	120.5 ± 12.33 ^b^	115.2 ± 10.11 ^a^	92.95 ± 4.26 ^c^
DSOR 17	84.14 ± 5.02 ^b^	111.5 ± 11.34 ^a^	132.50 ± 3.92 ^a^
DSOR 21	74.79 ± 5.90 ^b^	110.3 ± 9.30 ^a^	116.40 ± 4.22 ^a^
DSOR 24	112.9 ± 7.05 ^b^	118.2 ± 10.46 ^a^	98.56 ± 3.65 ^c^
DSOR 33	137.2 ± 12.45 ^a^	118.0 ± 9.13 ^a^	56.65 ± 4.71 ^b^

Results are expressed as mean ± SD. Different superscripts within each column denote significant difference (*p* < 0.05). DW, dry weight sample.

**Table 2:** Phenolic compounds identified in the ethanol extracts of DSOR 11 by UHPLC-Q-TOF-MS^2^. RT: Retention time.

Peak No.	RT (min)	Molecular Formula	Molecular Weight	[M−H]^−^ (*m*/*z*)	MS/MS (% Abundance)	Tentative Identification
1	8.86	C_30_H_26_O_12_	578.1428	577.1354	289 (92), 407 (62), 425 (55)	Procyanidin dimer
2	9.58	C_15_H_14_O_6_	290.0797	289.0719	109 (78), 123 (100), 203 (34)	(+)-Catechin
3	9.69	C_21_H_22_O_12_	466.1115	465.1042	275 (48), 285 (100), 303 (22)	Dihydromyricetin 3-*O*-rhamnoside
4	11.89	C_15_H_10_O_5_	270.0531	269.0459	117 (100), 149 (33), 227 (9)	Apigenin
5	12.26	C_16_H_12_O_5_	284.0690	283.0612	196 (100), 240 (88), 268 (32)	Glycitein
6	12.39	C_15_H_10_O_4_	254.0585	253.0508	117 (91), 210 (100), 225 (17)	Apigeninidin
7	12.49	C_15_H_12_O_7_	304.0587	303.0513	125 (100), 175 (24), 217 (8)	Taxifolin
8	12.69	C_16_H_12_O_4_	268.0743	267.0666	180 (100), 224 (91), 252 (34)	Formononetin
9	14.86	C_21_H_20_O_9_	416.1112	415.1037	135 (88), 161 (100), 253 (94)	1,3-*O*-dicaffeoylglycerol
10	15.56	C_15_H_12_O_5_	272.0686	271.0614	107 (38), 119 (100), 151 (30)	Naringenin
11	15.62	C_15_H_10_O_6_	286.0481	285.0406	133 (100), 151 (43), 217 (9)	Luteolin
12	16.49	C_15_H_10_O_5_	270.0531	269.0459	117 (100), 149 (33), 227 (9)	Apigenin

**Table 3:** Phenolic compounds identified in the ethanol extracts of DSOR 17 by UHPLC-Q-TOF-MS^2^. RT: Retention time.

Peak No.	RT (min)	Molecular Formula	Molecular Weight	[M−H]^−^ (*m*/*z*)	MS/MS (% Abundance)	Tentative Identification
1	9.58	C_15_H_14_O_6_	290.0797	289.0721	109 (78), 123 (100), 203 (34)	(+)-Catechin
2	9.64	C_21_H_22_O_12_	466.1115	465.1042	275 (48), 285 (100), 303 (22)	Dihydromyricetin 3-*O*-rhamnoside
3	9.67	C_24_H_22_O_14_	534.0988	533.0918	200 (20), 325 (100), 447 (3)	Luteolin 7-*O*-malonyl-glucoside
4	9.69	C_15_H_12_O_7_	304.0587	303.0513	125 (100), 175 (24), 217 (8)	Taxifolin
5	10.49	C_9_H_8_O_4_	180.0424	179.0350	117 (7), 135 (100)	Caffeic acid
6	12.41	C_15_H_12_O_7_	304.0587	303.0513	125 (100), 175 (24), 217 (8)	Taxifolin
7	12.45	C_15_H_10_O_6_	286.0481	285.0406	199 (100), 175 (65), 217 (26)	Kaempferol
8	12.69	C_16_H_12_O_4_	268.0743	267.0664	180 (100), 224 (91), 252 (34)	Formononetin
9	14.62	C_15_H_12_O_6_	288.0636	287.0562	125 (100), 151 (33), 193 (27)	Eriodictyol
10	14.86	C_21_H_20_O_9_	416.1112	415.1037	135 (88), 161 (100), 253 (94)	1,3-*O*-dicaffeoylglycerol
11	15.56	C_15_H_12_O_5_	272.0686	271.0614	107 (38), 119 (100), 151 (30)	Naringenin
12	15.63	C_15_H_10_O_6_	286.0481	285.0406	133 (100), 151 (43), 217 (9)	Luteolin
13	16.49	C_15_H_10_O_5_	270.0531	269.0457	117 (100), 149 (33), 227 (9)	Apigenin

**Table 4:** Phenolic compounds identified in the ethanol extracts of DSOR 33 by UHPLC-Q-TOF-MS^2^. RT: Retention time.

Peak No.	RT (min)	Molecular Formula	Molecular Weight	[M−H]^−^ (*m*/*z*)	MS/MS (% Abundance)	Tentative Identification
1	8.83	C_30_H_26_O_12_	578.1428	577.1355	289 (92), 407 (62), 425 (55)	Procyanidin dimer
2	9.58	C_15_H_14_O_6_	290.0797	289.0721	109 (78), 123 (100), 203 (34)	(+)-Catechin
3	9.64	C_21_H_22_O_12_	466.1115	465.1044	275 (48), 285 (100), 303 (22)	Dihydromyricetin 3-*O*-rhamnoside
4	9.67	C_24_H_22_O_14_	534.0988	533.0918	200 (20), 325 (100), 447 (3)	Luteolin 7-*O*-malonyl-glucoside
5	9.69	C_15_H_12_O_7_	304.0587	303.0513	125 (100), 175 (24), 217 (8)	Taxifolin
6	10.49	C_9_H_8_O_4_	180.0424	179.0350	117 (7), 135 (100)	Caffeic acid
7	10.45	C_21_H_22_O_12_	466.1115	465.1044	275 (48), 285 (100), 303 (22)	Dihydromyricetin 3-*O*-rhamnoside
8	12.24	C_16_H_12_O_5_	284.0690	283.0613	196 (100), 240 (88), 268 (32)	Glycitein
9	12.41	C_15_H_12_O_7_	304.0587	303.0513	125 (100), 175 (24), 217 (8)	Taxifolin
10	12.45	C_15_H_10_O_6_	286.0481	285.0406	199 (100), 175 (65), 217 (26)	Kaempferol
11	12.69	C_16_H_12_O_4_	268.0743	267.0664	180 (100), 224 (91), 252 (34)	Formononetin
12	13.34	C_15_H_12_O_7_	304.0587	303.0513	125 (100), 175 (24), 217 (8)	Taxifolin
13	14.86	C_21_H_20_O_9_	416.1112	415.1037	135 (88), 161 (100), 253 (94)	1,3-*O*-dicaffeoylglycerol
14	15.56	C_15_H_12_O_5_	272.0686	271.0615	107 (38), 119 (100), 151 (30)	Naringenin
15	15.69	C_15_H_10_O_6_	286.0481	285.0408	133 (100), 151 (43), 217 (9)	Luteolin
16	16.49	C_15_H_10_O_5_	270.0531	269.0459	117 (100), 149 (33), 227 (9)	Apigenin
17	16.55	C_16_H_12_O_6_	300.0638	299.0563	136 (16), 256 (33), 284 (100)	Hispidulin
18	17.76	C_16_H_12_O_6_	300.0638	299.0563	136 (16), 256 (33), 284 (100)	Hispidulin

**Table 5:** Quantification of polyphenolic compounds in decorticated sorghum extracts by high performance liquid chromatography photodiode array (HPLC-PDA).

Compound	RT (min)	DSOR 11 (µg/100 g)	DSOR 17 (µg/100 g)	DSOR 33 (µg/100 g)	Polyphenol Class
Gallic acid	2.40	ND	ND	ND	Phenolic acid
Catechin	9.58	103.03	83.55	140.99	Flavonoid
Caffeic acid	10.49	ND	44.93	49.82	Phenolic acid
*p*-Coumaric acid	12.06	ND	ND	ND	Phenolic acid
Ferulic acid	12.58	ND	ND	ND	Phenolic acid
Quercetin	15.38	ND	ND	ND	Flavonoid
Genistein	15.87	ND	ND	ND	Flavonoid

## Supplementary Materials

The following are available online, Table S1: Pearson correlation coefficients of total phenolic content, antioxidant capacities, digestive enzymes and AGE in decorticated sorghum ethanol extracts; Mass spectra data of individual phenolic compounds in negative electrospray ionization.
