# Supplementary material for: Flavonoids in Decorticated Sorghum Grains Exert Antioxidant, Antidiabetic and Antiobesity Activities
Source: Molecules. 2020 Jun 20;25(12):2854. doi: 10.3390/molecules25122854 (PMC7355972; doi:10.3390/molecules25122854)
Supplement: Supplementary file 1 [file molecules-25-02854-s001.zip › Supplementary data/Supplementary Table 1.doc]

**Supplementary Table 1.** Pearson correlation coefficients of total phenolic content, antioxidant capacities, digestive enzymes and AGE in decorticated sorghum ethanol extracts.

| Ethanol extracts | TPC | DPPH | ABTS | α-amylase | α-glucosidase | AGE | Lipase |
| --- | --- | --- | --- | --- | --- | --- | --- |
| DPPH | -0.77^*^ |  |  |  |  |  |  |
| ABTS | -0.47* | 0.63* |  |  |  |  |  |
| α-amylase | -0.65* | 0.69* | -0.05 |  |  |  |  |
| α-glucosidase | 0.33 | -0.15 | 0.15 | -0.46 |  |  |  |
| AGE | -0.07 | -0.18 | 0.08 | -0.23 | -0.37 |  |  |
| Lipase | -0.53 | 0.76 | 0.28 | 0.66 | 0.28 | -0.29 |  |

^*^Significant correlations (p ≤ 0.05).
